# Palliative Care e-Learning for Physicians Caring for Critically Ill and Dying Patients during the COVID-19 Pandemic: An Outcome Evaluation with Self-Assessed Knowledge and Attitude

**DOI:** 10.3390/ijerph191912377

**Published:** 2022-09-28

**Authors:** Jacqueline Schwartz, Manuela Schallenburger, Theresa Tenge, Yann-Nicolas Batzler, Daniel Schlieper, Detlef Kindgen-Milles, Stefan Meier, Günter Niegisch, André Karger, Christoph Roderburg, Martin Neukirchen

**Affiliations:** 1 Interdisciplinary Center for Palliative Medicine, University Hospital Duesseldorf, Medical Faculty, Heinrich Heine University Duesseldorf, 40225 Duesseldorf, Germany; 2 Department of Anesthesiology, University Hospital Duesseldorf, Medical Faculty, Heinrich Heine University Duesseldorf, 40225 Duesseldorf, Germany; 3 Department of Urology, University Hospital Duesseldorf, Medical Faculty, Heinrich Heine University Duesseldorf, 40225 Duesseldorf, Germany; 4 Institute for Psychosomatic Medicine and Psychotherapy, University Hospital Duesseldorf, Medical Faculty, Heinrich Heine University Duesseldorf, 40225 Duesseldorf, Germany; 5 Department of Gastroenterology, University Hospital Duesseldorf, Medical Faculty, Heinrich Heine University Duesseldorf, 40225 Duesseldorf, Germany

**Keywords:** palliative care, e-learning, education and training, symptom management, COVID-19, end-of-life care

## Abstract

During the COVID-19 pandemic, the care of critically ill and dying patients in isolation wards, intensive care units (ICUs), and regular wards was severely impaired. In order to support physicians in communicative and palliative care skills, an e-learning tool was developed as part of the joint project “Palliative Care in Pandemic Times” (PallPan). This study investigates the feasibility of this e-learning tool. Secondly, we aim to analyze changes in knowledge and attitude upon completion of the e-learning tool. A 38-item questionnaire-based evaluation study with assessment of global and specific outcomes including ICU and non-ICU physicians was performed. In total, 24 questionnaires were included in the anonymous analysis. Feasibility was confirmed by a very high rate of overall satisfaction (94% approval), with relevance reaching 99% approval. Overall, we detected high gains in knowledge and noticeably lower gains on the attitude plane, with the highest gain in naming reasons for incorporating palliative care. The lowest learning gain on the attitude plane was observed when the participants were confronted with their own mortality. This study shows that e-learning is a feasible tool for gaining knowledge and even changing the attitudes of physicians caring for critically ill and dying patients in a self-assessment evaluation.

## 1. Introduction

The COVID-19 pandemic has presented enormous challenges to health care workers [1]. In addition to the treatment of numerous severely ill and isolated patients with COVID-19 in intensive care units (ICUs) and regular wards, the care of critically ill and dying patients without COVID-19 was affected by the pandemic [2,3]. Increased hygienic standards; the fear of infection and virus spread; the lack of family visits, resulting in loneliness of patients at the end of their lives; and the overall threatening and uncertain situation affected patient care [3]. Additionally, staff communication skills were particularly critical during this time for conversations with patients, their next of kin (e.g., via video or telephone calls) as well as within and between interdisciplinary working teams. In this situation, university research groups from all over Germany founded a joint project named “National Strategy for Palliative Care of Severely Ill and Dying People and their Relatives in Pandemics” (PallPan) [4]. As part of this research group, the Interdisciplinary Center for Palliative Medicine in Duesseldorf conducted a qualitative study to investigate experiences and challenges related to the care of critically ill and dying patients (with or without COVID-19 infection) and their relatives in general inpatient palliative care (PC) [5]. From this study, six critical categories were identified, addressing the following topics: visiting regulations, communication with relatives, hygiene measures, cooperation, determination of the patients’ will, and the possibility of saying goodbye [5,6]. Based on our findings, an electronic learning (e-learning) tool was developed within the framework of PallPan to expand the scope of the results and offer interactive support to a larger number of health care workers [4,6]. Additionally, at the request of staff members of the interdisciplinary ICU at University Hospital Duesseldorf, the Interdisciplinary Center for Palliative Medicine created a support program with multi-professional communication seminars for health care workers [7]. These seminars provided relief for ICU staff through mutual interprofessional exchange and offered training in communication skills [7]. Experiences from previously conducted seminars were helpful to us in developing our e-learning tool. 

The e-learning tool consists of a free course comprising eight sections and targets staff of any professional group who are not working in the PC sector but still care for seriously ill patients during a pandemic. After a brief introduction, basic information on PC, such as common terms used in PC and symptom control, is provided. The following section focuses on communication between health care workers and patients or relatives, with special focus on digital communication tools. Further on, best practices concerning visiting regulations are discussed. This section is followed by an explanation of the importance of interprofessional collaboration. After the final section, which deals with supporting death and dying, the underlying PallPan study is described. The course ends with a voluntary quiz. Each section stands alone, and the course as a whole or a single section can be interrupted at any time. Videos showing communication between physicians and patients may be included. In addition, checklists (as a support tool for video calls, for example) provide quickly accessible support for everyday tasks. The e-learning tool is a voluntary program with no moderator or a specific educational goal, besides educating oneself and gaining confidence in dealing with seriously ill patients. Furthermore, no evaluation or follow-up for participants takes place. The e-learning tool is designed to offer useful knowledge for everyday tasks in an easily accessible fashion.

E-learning in general can be provided in real-time via an online seminar or as an individual course depending on the participant’s availability. We selected the latter asynchronous model, which allows participants to complete the course at their own pace and the instructor to record the content only once. However, with this model, participant motivation and spare time are absolutely required [8]. The positive effects of e-learning courses in terms of accessibility, efficacy, cost effectiveness, and flexibility are summarized in the review by Sinclair [9]. 

E-learning formats have also gained importance for PC. The European Association for Palliative Care (EAPC) supports PC education through shared learning opportunities [10]. Furthermore, EAPC highlights that using digital learning tools enhances the understanding of PC theory and its application to practice [10]. Gibbins showed that e-learning for medical students changes attitudes on “palliative thinking” [11]. Until now, many studies have only involved medical students in research questioning the use of e-learning in PC [11,12,13,14,15]. A mixed-methods study asking all PC health care workers in Ireland to name factors that motivate participation in e-learning revealed the following responses: dedicated time to participate in e-learning activities, quick technical and administrative support, computer training before completing an e-learning course, and regular contact with the tutor in online course work [8]. In Ontario, Canada, during the COVID-19 pandemic, a virtual educational tool to prepare physicians for making and communicating difficult triage decisions was developed and has led to improved confidence and knowledge [16].

Studies show that e-learning can be an easy and low-threshold tool for gaining competence. At present, to our knowledge, there is no study regarding the effects of e-learning on physicians who are not working in PC but caring for seriously ill or dying patients during the COVID-19 pandemic. The objective of this study is a global and outcome-based evaluation of the PallPan e-learning tool. We aim to investigate (1) the feasibility and (2) the impact on the (a) knowledge and (b) attitude of physicians caring for critically ill and dying patients in a pandemic.

## 2. Materials and Methods

This study is a prospective single-center study conducted at University Hospital Duesseldorf, Germany. Ethical approval was obtained by the local ethics committee (reference number 2022-1906). The PallPan e-learning tool is available online (www.pallpan.de/elearning, accessed on 9 March 2021) free of charge as of November 2021 and takes approximately 60 min to complete. The content is exclusively in German. 

Participation in the study was completely anonymous and voluntary and could be withdrawn at any time without explanation. The e-learning site does not save any personal data and can be freely accessed. For the online survey, the saving of IP addresses was blocked so that no tracing was possible. In the case of drop-outs, the data were saved but automatically extracted prior to analysis to ensure that the data were not used. The inclusion criteria were as follows: age > 18 years; physician with direct contact with critically ill and dying patients during the COVID-19 pandemic; a fully completed questionnaire; and informed consent to participate in the study. Before starting the online questionnaire, the participants were asked to confirm via a check box that they had read and understood the written informed consent form concerning ethics and data protection and accepted the rules. Without this confirmation, participation was not possible. Due to the open request for participation, calculation of the response rate is not possible.

### 2.1. Measures

The structured questionnaire, which is based on preliminary work by the working group [14,17] and was developed in interdisciplinary and multi-professional discussions in the Interdisciplinary Center for Palliative Care Medicine, is written in German and can be found in the Supplementary Materials (translated into English, Questionnaire). In total, the questionnaire comprised 42 statements or questions covering three areas: an outcome-based evaluation, a global evaluation and demographic data. Part 1 contained 24 statements focused on knowledge and attitude (Table 1) based on the ten core competencies defined by the European Association of Palliative Care in 2013 [10]. Part 2 consisted of 14 statements that judged the feasibility of the e-learning tool, ranking items such as structure, efficiency, effectiveness, comprehensiveness, and satisfaction. Finally, four questions covered demographic data, asking for age, gender, work experience, and experience in intensive care medicine. The global outcome evaluation on feasibility utilized a 5-point Likert scale (from fully agree to strongly disagree), where “fully agree” and “strongly agree” were considered agreement. For the specific outcome evaluation, the German school grading system (1 = “excellent” to 6 = “unsatisfactory”) was used as a rating scale. The survey was to be conducted after completion of the e-learning, whereby the specific outcome evaluation was conducted retrospectively in a post-then method. Anonymized data collection was performed by using the online survey platform Unipark [18].

### 2.2. Data Analysis

Data analysis was performed using Microsoft Excel 2020 (version 16.42, Microsoft Corp., Redmond, WA, USA) and IBM SPSS Statistic version 28.0.1.1 (IBM, Armonk, NY, USA). For the global evaluation concerning feasibility, a descriptive analysis (frequencies in percent) was conducted. For the outcome-based evaluation, the participants assessed their learning progress by self-evaluating their skills before and after completion of the e-learning tool retrospectively. This method was also utilized in previous studies [12,14], whereby the advantage of the calculation is that previous experiences of the participants, here, e.g., palliative previous experiences, are factored out and thus have no influence on the results. Subsequently, the learning gain can be measured as the “Comparative Self-Assessment Gain” [20]:

CSA Gain [%] = ((µ_pre_ − µ_post_)/(µ_pre_ − 1)) × 100. (1)

CSA gain was calculated with a 95% confidence interval and standard error using individual learning gain (ILG) values. These values were calculated using the formulas (1) ILG = 0 for pre = post and (2) ILG = (pre − post)/(pre − 1) × 100 for pre > post. Participants with missing values were excluded from the individual item.

## 3. Results

From April to June 2022, a total of 80 physicians (35 intensivists, 34 gastro-oncologists, and 11 uro-oncologists) were contacted and given general information about the study as well as direct, non-personalized links to the e-learning course and the questionnaire. A total of 46 (57.5%) physicians participated in the PallPan e-learning tool and 24 (30%) completed the questionnaire.

### 3.1. Descriptive Statistics

Most participants were between 35 and 44 years of age with a balanced gender ratio. Work experience in general was more than five years, and 71% of the participants had experience in intensive care. The demographic data are shown in Table 2.

### 3.2. Global Evaluation—Feasibility

Overall, the e-learning tool received very positive ratings. In total, 96% of the participating physicians were satisfied with the tool and all participants felt that it was useful for their work. With regard to the specific content, 92% (statement 11) and 96% (statement 3) found the e-learning tool to be comprehensive. Other items such as structure, effectiveness and efficiency also received very positive ratings with over 83% agreement for the various statements. For details see Table 3.

### 3.3. Outcome-Based Evaluation—Effect on Knowledge and Attitude

**Figure 1:**
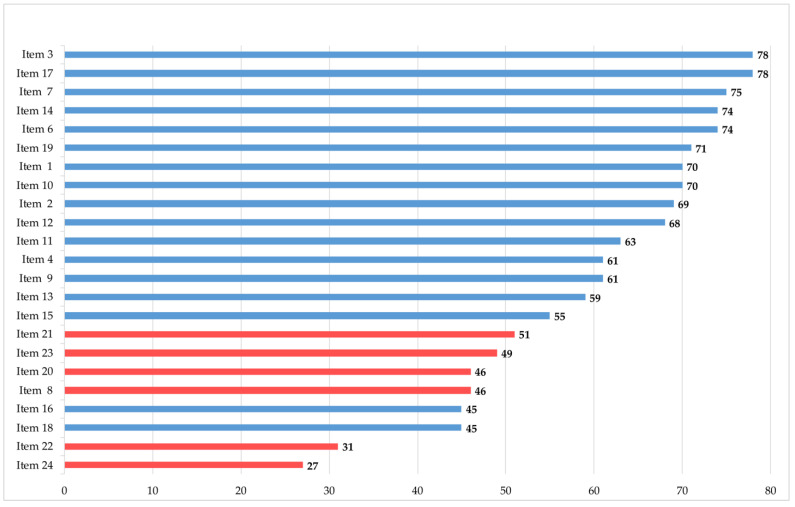
Comparative Self-Assessment (CSA) gains in %. Items included knowledge (blue) and attitude (red).

In the outcome-based evaluation, higher learning gains were detected on the knowledge plane than on the attitude plane (Figure 1). The highest learning gains on the knowledge plane were observed in naming reasons for incorporating PC, being aware of the importance of interprofessional and interdisciplinary collaboration and understanding the importance of relatives and their care (CSA gain 78%, 78%, and 75%, respectively). The lowest learning gains were detected on the attitude plane in dealing with their own mortality (CSA gain 27%), reflecting their own attitude on death and dying (CSA gain 31%) and supporting patients and relatives in saying goodbye (CSA gain 45%). For further details, see Figure 1.

Statistical analysis using 95% confidence intervals confirmed the learning gains, which were significant for 23 items. Item 24, which has the lowest CSA gain had no significant learning gain (Table 4). There was no significant difference in CSA gains between experienced (>10 years’ work experience) and inexperienced (<10 years’ work experience) physicians.

According to the overall findings, we detected higher learning gains on the knowledge plane than on the attitude plane in the subgroup of physicians with experience in intensive care medicine (71% of the participants) as well. We had comparable results for the lowest CSA gain, whereas the highest CSA gain was found in familiarity with the term palliative sedation. In general, we detected higher CSA gains in most items (except Items 2, 12 and 19) compared to physicians without experience in intensive care medicine. Palliative experience was not measured as it is factored out with the CSA gain method and has no influence.

## 4. Discussion

Due to pandemic restrictions, educational offerings for physicians such as conferences or face-to face courses were cancelled, leading to an “educational gap.” At the same time, a growing number of seriously ill and dying patients with and without COVID-19 underlined the need for education despite the limited time available. To close this gap, we developed an e-learning tool within the PallPan framework. In our presented study, we investigated whether the tool was feasible and if changes in the knowledge and attitude of physicians caring for seriously ill and dying patients were achievable via an e-learning program. 

During the COVID-19 pandemic, a growing number of e-learning programs were established and investigated, but these were mostly intended as a substitute for in-person education. However, e-learning data, especially for programs targeting health care workers in a pandemic, are scarce. The existing literature includes, for example, the feasibility of blended learning formats for paramedics preparing for work in ICUs [21]. To our knowledge, the presented data are the first to evaluate e-learning for pandemic-focused PC. 

In general, health-related e-learning programs provide increased accessibility to education, efficacy, cost effectiveness, and learner flexibility [9]. Additionally, the usefulness of e-learning in enhancing knowledge has been previously shown [22]. To evaluate e-learning, Kirkpatrick established a four-level model [23]. Based on this model, we inquired about satisfaction (Level 1) in the descriptive analysis and evaluated knowledge acquisition (Level 2). Due to the e-learning format, which focuses on teaching knowledge, we are not able to evaluate learning gains in skills or changes in clinical behavior, which represent Levels 3 and 4 of Kirkpatrick’s model [23]. In this context, adaption of the e-learning program and re-evaluation—after several weeks for instance—would be interesting. It would be worthwhile to determine if self-reported changes translate to changes in clinical behavior. As we were able to show, the e-learning program achieved very high ratings both in terms of satisfaction as well as in providing a useful and comprehensive tool for deepening or refreshing knowledge of PC. Consistently, high approval rates in all subcategories, such as overall satisfaction, structure, effectiveness, comprehensiveness, and efficiency, underline the feasibility of the e-learning tool. The highest approval rating (99%) was found in “usefulness for my work,” which clearly indicates the content’s relevance. 

The need for multidisciplinary PC teams to support physicians in regular or intensive care wards is well known [24]. Especially in a pandemic with high numbers of seriously ill or dying patients [25], specialist palliative care professionals may not be able to conduct all the consultations required. In those cases, or as in some German hospitals where there are no specialist PC professionals at all, e-learning is an effective means to support patient care for non-specialized physicians. 

As mentioned above, the EAPC recommends using digital learning to enhance understanding of PC theory and its application to practice [10]. E-learning or blended courses to teach PC subjects are offered [26] and evaluated worldwide [27,28]. However, few data evaluate gains in crucial competencies for providing PC, such as knowledge, attitude, or communication skills [28,29]. In the evaluated courses, blended learning formats were frequently used to address skills and changes in clinical behavior with an evaluation several weeks or months later. The PallPan e-learning program is not a blended format and does not offer real-time interaction with a teacher. Therefore, our evaluation focused on knowledge gain and sought to determine whether and to what extent changes in PC attitudes are achievable. As we were able to show, e-learning is effective in increasing knowledge on a high level in a self-assessment evaluation, with CSA gains between 45% and 78%. If we look at the different items in detail, higher learning gains are detected when only knowledge is taught, such as in naming reasons for incorporating PC (Item 3, CSA gain 78%) or defining palliative sedation (Item 6, CSA gain 74%). The learning gains for items associated with possible skills or clinical behavior are lower (e.g., Item 18: supporting patients and relatives in saying goodbye). This comes as no surprise, as the latter involves not only knowledge but also certain skills, which require practice and experience to be improved [30]. 

The lowest CSA gains were detected on the attitude plane, especially for those items that deal with the participants’ own mortality or fear of death (Item 22 and 24). These items are existential in nature and require the participant’s willingness to engage. Even though the responses are determined by the participant’s individual beliefs and experiences, e-learning without interaction or discussion might still evoke a change in attitude [31]. 

### Study Limitations

This study had several limitations. First, PC is known to be a multidisciplinary holistic field of work, and we usually intend to address other professions as well. PallPan itself addresses all professions that care for severely ill and dying patients, including not only physicians and nurses but also social workers, physiotherapists, chaplains, etc. Due to the current and ongoing shortage of nurses in the German health care system, we decided to focus our survey solely on physicians. Here, further surveys are needed to recognize probable differences among specific professional groups. Secondly, the number of participants is rather small, the sample is not representative and the questionnaire has not been piloted or previously validated. Furthermore, we did not record whether the participants completed all parts of the e-learning course before answering the questionnaire. The CSA gain is completely reliant on self-assessment. Furthermore, it was not possible to check whether the participants completed the questionnaire directly after finishing the e-learning. A possible time gap could have influenced the memory and, thus, the self-assessment. Moreover, there was no control group.

## 5. Conclusions

Our evaluation shows that e-learning is a successful tool for gaining knowledge and even generating changes in the attitudes of physicians caring for critically ill and dying patients in a self-assessment evaluation. Especially in a pandemic with limited educational offerings and a shortage of health care workers, our e-learning tool provides useful support for physicians who treat patients with life-limiting diseases.

## Figures and Tables

**Table 1:** Statements.

No.	Statement	Competency
1	I am familiar with the four dimensions of palliative care.	Knowledge
2	I am able to explain different models of palliative care delivery.	Knowledge
3	I am capable of naming reasons for incorporating specialized palliative care into standard care.	Knowledge
4	I am aware of symptoms such as dyspnea/cough, restlessness/anxiety, and death rattle and can name interventions to relieve them.	Knowledge
5	I know to use opioids to treat dyspnea (substance, dose, interval, administration).	Knowledge
6	I am familiar with palliative sedation and am able to explain its meaning.	Knowledge
7	I understand the importance of relatives and their care.	Knowledge
8	I can critically reflect on my own strengths and weaknesses in conducting conversations.	Attitude
9	I know the SPIKES [19] model and can use it in conversations.	Knowledge
10	I know how to conduct digital conversations with relatives.	Knowledge
11	I know different methods of digital communication, can implement them, and can name best practice examples.	Knowledge
12	I know how to replace facial expressions with other communication methods when wearing a face shield.	Knowledge
13	I know how to talk to patients about changing and adjusting treatment goals.	Knowledge
14	I know that treatment decisions require a medical indication as well as the patient’s or the authorized representative’s consent.	Knowledge
15	I know how to talk to patients about the triage process.	Knowledge
16	I can recognize to what extent patient goals are appropriate and achievable.	Knowledge
17	I know the importance of interprofessional and interdisciplinary collaboration.	Knowledge
18	I am able to support relatives and patients in saying goodbye.	Knowledge
19	I am familiar with how tasks are performed in a palliative care ward.	Knowledge
20	I can interact with severely ill patients without fear.	Attitude
21	I can interact with people at the end of their life knowing that I cannot fully understand their specific situation.	Attitude
22	I am able to reflect on my own attitude toward death and dying.	Attitude
23	Interacting with dying patients and their relatives forces me to confront my own mortality.	Attitude
24	I am able to deal with my own mortality.	Attitude

**Table 2:** Demographic data of study participants (*n* = 24).

Factor	Answers, *n* (%)					
Age	<25 y	25–34 y	35–44 y	45–54 y	55–64 y	>64 y
	1 (4)	8 (33)	9 (37)	5 (20)	1 (4)	0 (0)
Gender	Male	Female	Other			
	14 (58)	10 (42)	0 (0)			
Work experience	<5 y	5–9 y	>10 y			
	5 (22)	7 (30)	11 (48)			
Experience in intensive care medicine	yes	no				
	17 (71)	7 (29)				

**Table 3:** Feasibility and agreement in %.

No.	Statement	Factor	Agreement
1	The e-learning tool was user-friendly.	Structure	96%
2	The amount of time needed to complete the e-learning program was reasonable.	Efficiency	83%
3	The e-learning tool is suitable for deepening/consolidating knowledge about caring for seriously ill and dying patients in a pandemic.	Comprehension	96%
4	The content of the e-learning tool is clearly presented.	Structure	96%
5	The e-learning tool is visually appealing.	Structure	96%
6	The e-learning tool is relevant to my work.	Effectiveness	92%
7	The e-learning tool is useful for my work.	Effectiveness	100%
8	The “checklist telephone call” is a helpful tool that I will use in the future.	Effectiveness	88%
9	The recommendation “180 s/6 items” is a helpful tool that I will use in the future.	Effectiveness	88%
10	The videos help me transfer my knowledge to daily tasks.	Structure	83%
11	The important issues in dealing with severely ill or dying patients are addressed completely.	Comprehension	92%
12	The layout is useful for targeting specific topics.	Structure	96%
13	I will use the e-learning tool for specific questions in the future.	Satisfaction	84%
14	Overall, I am satisfied with the e-learning tool.	Overall Satisfaction	96%

**Table 4:** 95% Confidence Interval (CI) and Standard Error (SE) for all Items.

Factor	*n*	95% CI	SE (%)
1	18	0.37–0.74	0.087
2	20	0.22–0.62	0.095
3	20	0.30–0.77	0.112
4	20	0.20–0.57	0.089
5	20	0.22–0.62	0.095
6	20	0.34–0.73	0.092
7	20	0.32–0.78	0.108
8	20	0.18–0.55	0.088
9	20	0.25–0.64	0.093
10	20	0.48–0.74	0.063
11	20	0.41–0.66	0.058
12	20	0.36–0.72	0.086
13	20	0.24–0.61	0.088
14	19	0.08–0.52	0.105
15	19	0.32–0.64	0.077
16	19	0.18–0.54	0.084
17	19	0.09–0.55	0.110
18	19	0.16–0.49	0.077
19	19	0.20–0.64	0.103
20	19	0.10–0.42	0.077
21	19	0.13–0.53	0.095
22	19	0.01–0.29	0.065
23	19	0.02–0.30	0.067
24	19	−0.04–0.20 **	0.058

** not significant.

## Data Availability

Not applicable.

## Supplementary Materials

The following supporting information can be downloaded at https://www.mdpi.com/article/10.3390/ijerph191912377/s1, Questionnaire.
