# Supplementary material for: Palliative Care e-Learning for Physicians Caring for Critically Ill and Dying Patients during the COVID-19 Pandemic: An Outcome Evaluation with Self-Assessed Knowledge and Attitude
Source: Int J Environ Res Public Health. 2022 Sep 28;19(19):12377. doi: 10.3390/ijerph191912377 (PMC9564513; doi:10.3390/ijerph191912377)
Supplement: Supplementary file 1 [file ijerph-19-12377-s001.zip › Questionnaire final.pdf]

UKD Universitätsklinikum Düsseldorf

Interdisciplinary Center for Palliativ Medicine (IZP) - University Hospital Düsseldorf - Moorenstraße 5 - 40225 Düsseldorf

Questionnaire:
CoViD-19: A survey of the feasibility and effect on knowledge and attitude of the „PallPan" e-learning tool among health care workers in the care of critically ill and dying patients in pandemic times.

Filling in the survey takes about 20 minutes.

Please consider two points in time:
Pre = retrospective self-assessment before starting the eLearning tool.
Post = self-assessment after completing the eLearning tool.

	**Part 1: Knowledge and Attitude**								
	Please respond to the following statements by rating them according to the German school grading system (1 = very good - 6 = unsatisfactory).								
	**Statement**		**Rating**						
				1	2	3	4	5	6
1	I am familiar with the four dimensions of palliative care		**Pre**						
			**Post**						
2	I am able to explain different models of palliative care delivery		**Pre**						
			**Post**						
3	I am capable naming reasons for incorporating specialized palliative care into standard care		**Pre**						
			**Post**						
4	I am aware of symptoms such as dypnoea/cough, restlessness/anxiety, and death rattle and can name interventions to relieve them		**Pre**						
			**Post**						
5	I know to use opioids to treat dyspnea (substance, dose, interval, administration)		**Pre**						
			**Post**						

6	I am familiar with palliative sedation and I am able to explain its meaning		**Pre**						
			**Post**						
7	I understand the importance of relatives and their care		**Pre**						
			**Post**						
8	I can critically reflect on my own strengths and weaknesses in conducting conversations.		**Pre**						
			**Post**						
9	I know the SPIKES model and can use it in conversations		**Pre**						
			**Post**						
10	I know how to conduct digital conversations with relatives		**Pre**						
			**Post**						
11	I know different ways of digital communication, can implement them and name best practice examples		**Pre**						
			**Post**						
12	I know how to replace facial expressions with other communication methods when wearing a face shield		**Pre**						
			**Post**						
13	I know how to talk to patients about changing and adjusting treatment goals		**Pre**						
			**Post**						
14	I know that treatment decisions require a medical indication as well as the patient`s or the authorized representative`s consent.		**Pre**						
			**Post**						
15	I know how to talk to patients about the triage process		**Pre**						
			**Post**						
16	I can recognize to what extent patient goals are appropriate and achievable		**Pre**						
			**Post**						

17	I know the importance of interprofessional and interdisciplinary collaboration.		**Pre**						
			**Post**						
18	I am able to support relatives and patients saying goodbye		**Pre**						
			**Post**						
19	I am familiar with how tasks are performed in a palliative care ward		**Pre**						
			**Post**						
20	I can interact with severely ill patients without fear		**Pre**						
			**Post**						
21	I can interact with people at the end of their life, knowing that I cannot fully understand their specific situation.		**Pre**						
			**Post**						
22	I am able to reflect my own attitude toward death and dying		**Pre**						
			**Post**						
23	Interacting with dying patients and their relatives forces me to confront my own mortality.		**Pre**						
			**Post**						
24	I am able to deal with my own mortality.		**Pre**						
			**Post**						

**Part 2: Feasibility**							
	Please rate the following statements by using the provided scale						
	**Statement**		**Rating**				
			**Strongly agree**	**Agree**	**Rather agree**	**Undecided**	**Rather disagree**
1	The e-learning tool was user-friendly						
2	The amount of time needed to complete the e-learning program was reasonable						

3	The e-learning tool is suitable for deepening/consolidating knowledge about caring for seriously ill and dying patients in a pandemic						
4	The content of the e-learning tool is clearly presented						
5	The e-learning tool is visually appealing						
6	The e-learning tool is relevant to my work						
7	The e-learning tool is useful for my work						
8	The “checklist telephone call” is a helpful tool that I will use in the future.						
9	The recommendation “180 seconds/6 items” is a helpful tool that I will use in the future						
10	The videos help me transfer my knowledge to daily tasks						
11	The important issues in dealing with severely ill or dying patients are addressed completely						
12	The layout is useful for targeting specific topics						
13	I will use the e-learning tool for specific questions in the future						
14	Overall, I am satisfied with the e-learning tool						

Part 3: Demografic Data

Please mark the correct answer.

1. **How old are you?**
   ❑ <25y. ❑ 25-34y. ❑ 35-44y. ❑ 45-54y. ❑ 55-64y. ❑ >64y.

2. **What gender do you identify as?**
   ❑ Female ❑ Other ❑ Male

3. **Do you have experience in intensive care medicine?**
   ❑ Yes ❑ No

4. **How many years of work experience do you have?**
   ❑ <5y. ❑ 5-9y. ❑ >10 y.
